# Accurate Coregistration between Ultra-High-Resolution Micro-SPECT and Circular Cone-Beam Micro-CT Scanners

**DOI:** 10.1155/2010/654506

**Published:** 2010-10-05

**Authors:** Changguo Ji, Frans van der Have, Hugo Gratama van Andel, Ruud Ramakers, Freek Beekman

**Affiliations:** ^1^Image Sciences Institute and Rudolf Magnus Institute, University Medical Center Utrecht, Universiteitsweg 100, 3584 CG Utrecht, The Netherlands; ^2^Physics Department, Peking University, Beijing 100871, China; ^3^MILabs B.V., Heidelberglaan 100, 3584 CX Utrecht, The Netherlands; ^4^Department of Radiation, Radionuclides, and Reactors, Section Radiation Detection and Medical imaging, Delft University of Technology, Mekelweg 15, 2629 JB Delft, The Netherlands

## Abstract

*Introduction*. Spatially registering SPECT with CT makes it possible to anatomically localize SPECT tracers. In this study, an accurate method for the coregistration of ultra-high-resolution SPECT volumes and multiple cone-beam CT volumes is developed and validated, which does not require markers during animal scanning. *Methods*. Transferable animal beds were developed with an accurate mounting interface. Simple calibration phantoms make it possible to obtain both the spatial transformation matrix for stitching multiple CT scans of different parts of the animal and to register SPECT and CT. The spatial transformation for image coregistration is calculated once using Horn's matching algorithm. Animal images can then be coregistered without using markers. *Results*. For mouse-sized objects, average coregistration errors between SPECT and CT in *X*, *Y*, and *Z* directions are within 0.04 mm, 0.10 mm, and 0.19 mm, respectively. For rat-sized objects, these numbers are 0.22 mm, 0.14 mm, and 0.28 mm. Average 3D coregistration errors were within 0.24 mm and 0.42 mm for mouse and rat imaging, respectively. *Conclusion*. Extending the field-of-view of cone-beam CT by stitching is improved by prior registration of the CT volumes. The accuracy of registration between SPECT and CT is typically better than the image resolution of current ultra-high-resolution SPECT.

## 1. Introduction

SPECT and CT are complementary in the sense that SPECT visualizes functional tracers at a very low concentration and CT shows anatomy that can be used to localize tracer activity with respect to tissue structures. Various rail-based, docking, and transferable approaches have been proposed for combining anatomical and nuclear medicine images, for humans as well as for small animals [1]. Compared with integrated devices such as SPECT-CT and PET-CT, the modular imaging modality set-ups can help to reduce costs, facilitate using modalities in parallel, and enable the replacement and upgrade of individual modalities [1–5]. Being an important tool in the field of multimodality imaging, image registration has been studied extensively [6, 7]. Accurately co-registered images of complementary imaging devices, for example, images representing tissue function (e.g., PET or SPECT) registered with structural/anatomical images, are of crucial importance to biomedical research. Firstly, multimodal images make it possible to accurately localize tracers at, for example, the site of a tumor or infection process in a specific tissue or organ [8]. Secondly, they can be used to enhance tracer quantification [9–11]. At the same time, in preclinical studies, a fast and accurate way to combine small-animal PET or SPECT images with CT or MRI images is required. The logistics of transferring animals between modalities are different compared to the clinical situation. The use of anesthesia, together with mild fixation (e.g., simple taping), virtually guarantees the animal stays in a stable position throughout the different scans. 

Hardware registration of preclinical imaging by a predetermined spatial transformation has been studied to combine separate stand-alone systems [12, 13]. Jan et al. registered the micro-PET-CT-SPECT whole-body images using a rigid-body transformation and a dedicated mouse-holder, whereby a phantom containing three line sources was used to determine the transformation matrix [12]. The manual alignment of the holder is deemed as the main source of registration errors.

Chow et al. registered micro-PET and micro-CT images using a 15-parameter perspective model [13]. In their work the spatial transformation matrix is obtained by a sophisticated grid phantom with 1288 lines to account for variations in CT voxel size due to imperfect alignment of the X-ray detector relative to the X-ray source. 

In this paper we developed and validated a method to automatically and accurately register micro-SPECT volumes with multiple circular cone-beam CT volumes. The method uses a phantom with a limited number of point sources to calculate a rigid-body transformation. We found that a more complicated transformation, as used by Chow et al. [13], is not necessary as the variations in voxel size in the used CT system are limited. To minimize the registration errors introduced by transferring of the animal from one modality to the other, as seen in Jan et al. [12], dedicated animal beds and bed-scanner interfaces were developed. The resulting method was tested in a number of image registration experiments.

## 2. Materials and Methods

### 2.1. Imaging Instruments

The SPECT device used (U-SPECT-II, Milabs BV, The Netherlands) has a high mechanical stability because it uses stationary detectors [14–19]. The transferable animal bed can be easily and rapidly mounted on an *X* *Y* *Z* stage, which translates the animal during scanning. The differently sized cylindrical collimators, each containing 75 pinhole apertures, are interchangeable to enable both mice and rats to be optimally scanned. A graphical user interface, which incorporates the preselection of the field-of-view using integrated optical cameras [20], allows the system to be focused on any volume-of-interest. This minimizes the scanning time and maximizes sensitivity. SPECT images are iteratively computed using pixel-based ordered-subset expectation maximization (POSEM [21]) implemented in a multithreaded way. 

The fast circular cone beam X-ray CT system used in this study (U-CT, Milabs BV, The Netherlands) is a dedicated tool for high-throughput low dose in vivo scanning of small animals. It uses an air-cooled metalloceramic tube with a voltage range of 20–65 kV and a 1280 × 1024 pixel, 12-bit semiconductor digital camera as a detector. The scanning volume is 82 mm in diameter, 82 mm single scan length, and 212 mm full scan length. The voxel size (isotropic) is 83 *μ*m or 166 *μ*m.

### 2.2. Animal Bed-Mounting

**Figure 1:**
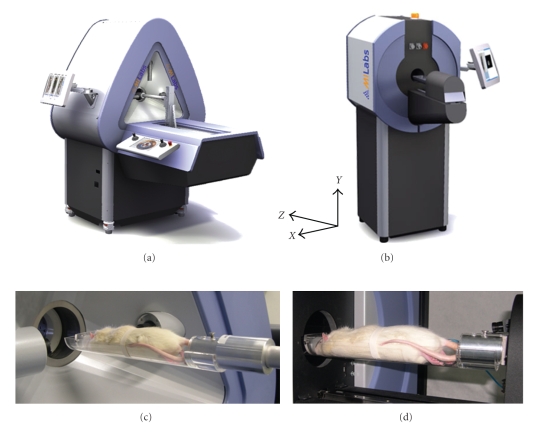
Modular approach to combine small-animal SPECT and CT scanners with transferable animal beds. (a) Micro-SPECT system (U-SPECT-II). (b) X-ray CT system (U-CT). (c) Rat bed on U-SPECT-II scanner. (d) Rat bed on U-CT scanner. Inset shows coordinate axes.

Different sizes of transferable animal beds have been developed for mice and rats. They are designed in such a way that they can be quickly, easily, but also precisely and reproducibly mounted and demounted from the SPECT and CT scanners. As the beds are made from polymethylmethacrylate (PMMA) material, it can be assumed that this method can be generally applied to other imaging systems, such as MRI and PET. The SPECT system automatically detects the type and size of the bed by means of switches. To mount the bed to the robot arm, the bed is locked in place during scanning. It can be quickly unlocked and transferred to other imaging modalities without disturbing the relative position of the animal (see Figure 1).

**Figure 2:**
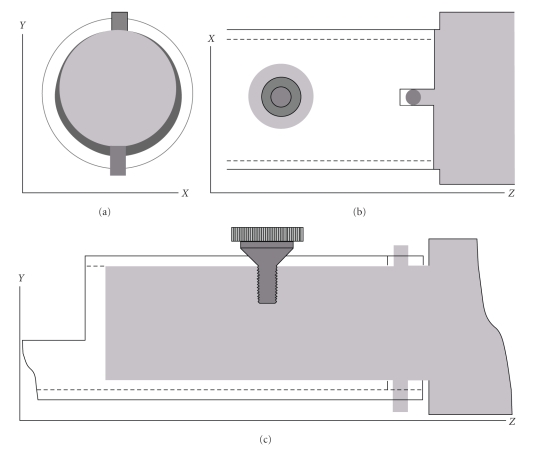
Method of bed-mounting: (a) *Y* *X* plane (Cross-section), (b) *X* *Z* view, and (c) *Y* *Z* view.

Figure 2 shows that the end of the bed is a hollow cylinder that moves over a cylindrically shaped metal extension connected to the robot arm (grey). This allows for a relatively large play between the robot arm and cylindrical bed end. It ensures that the bed can be easily and rapidly mounted and demounted. Reproducibility of bed positioning is guaranteed by means of a conical screw restricting the motion of the bed in the *X* and *Z* directions, while in the *Y* direction there is a line of contact between the bed and the robot arm on the top side. Two additional pins (also in grey, frames (b) and (c)) restrict the rotation around the axis of the screw and around the line of contact.

### 2.3. Voxel Size Calibration

**Figure 3:**
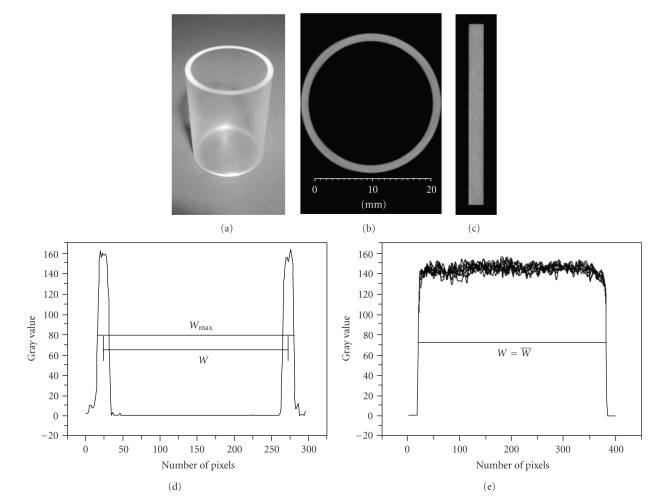
Reconstructed cylinder images and profiles used to calculate CT voxel size. (a) Cylinder made of polyethylene. (b) Cross-sectional image used to determine voxel size in the *X* and *Y* direction. (c) The longitudinal section image used to determine the voxel size in the *Z* direction. (d) Profile and line segment used to determine voxel size in the *X* and *Y* direction. (e) Profile and line segment used to determine voxel size in the *Z* direction.

For SPECT images, the voxel size is precisely defined during the measurement of the system matrix for image reconstruction [15]. To determine the CT voxel size, a polyethylene cylinder with its outer diameter, inner diameter, and length precisely known (±0.01 mm) was machined (Figure 3(a)). The cylinder was scanned with its axial direction aligned to the *Z* direction. After reconstruction, the axis of the reconstructed cylinder was further aligned to the *Z*-axis of the CT reconstruction volume. In order to calibrate the *X* and *Y* voxel size, we established a line in which the outer diameter was at its maximum. A profile was then drawn. From the profile (see Figure 3(d)), the FWHM values representing the inner and outer diameter were determined. The average value of *W *was then used to calculate the voxel size in the *X* and *Y* directions. The average FWHM value of the cylinder height was used to determine the voxel size in the *Z* direction (see Figure 3(e)).

**Figure 4:**
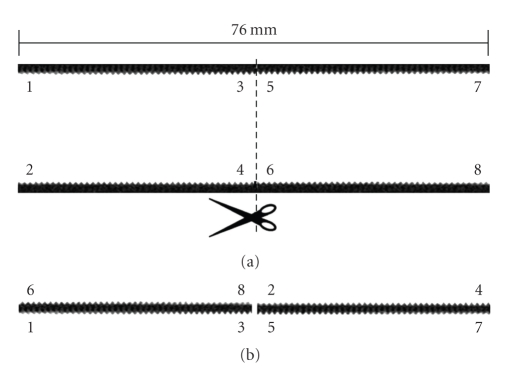
Micro-CT images of a cylinder with regular screw thread on the surface. (a) Cross-section image of the whole FOV. (b) Rearranged Cross-section image of different parts of frame A, in such a way that the central part of the FOV from one side (e.g., 3, 5) is displayed opposing the peripheral part of the FOV from the other side of the cylinder (e.g., 8, 2). There is a one-to-one correspondence in the screw thread on both sides of the interface in frame (b), therefore any voxel size variations between central and peripheral parts must be small.

In order to establish whether there are variations in voxel size large enough that they might influence the coregistration accuracy based on a rigid transformation, we used a solid cylinder (PMMA, diameter 20 mm) with a screw thread (1 mm interval) on the surface. Variations in reconstructed voxel size over the field-of-view might be expected because of the cone-beam geometry, where the reconstructed voxel size could be expected to vary in the axial direction from the peripheral parts of the field-of-view to the central part of the field-of-view. A micro-CT scan of the cylinder was made, where the axial direction of the cylinder was aligned exactly to the *Z* direction of the reconstruction volume. The reconstruction was cut in two halves (from one side peripheral to central and from central to peripheral on the other side). The reconstructed volume is shown in one piece in Figure 4(a) and rearranged in Figure 4(b), where the central part of one half is matched to the peripheral part of the other. If the voxel size would vary, there would not be a one-to-one correspondence in the regular screw thread between the two parts. Although some artifacts can be observed in the peripheral parts of the FOV due to the cone-beam geometry, no irregularities can be observed in the correspondence of the screw thread between the two halves on the surface (see Figure 4). Since there is a one-to-one match, which does not deviate more than a quarter of the thread distance, the expected position measurement errors introduced by voxel size variations are small enough to justify the use of a rigid-body transformation method.

### 2.4. Registration Phantom

We designed special mouse and rat registration phantoms with point sources as markers to calculate the spatial transformation using a rigid-body model. The sizes of the phantoms are chosen such that they are about the same as the body size of the animals. This is 20 × 20 × 70 mm^3^ for mouse imaging and 36 × 36 × 150 mm^3^ for rat imaging. The point sources are made by evaporating a fluid containing both contrast agent (Iopromide, visible in CT, 150 mg I/mL) and activity (^99 m^Tc, visible in SPECT, 600 MBq/mL) on ion-exchange resin beads (AG 1-X2, 50–100 Mesh, 180–500 *μ*m wet bead size, Bio-Rad) in a small cup. The volume percentages of each in the mixture are, respectively, 5% and 95%. The beads are placed in the mixture on a heating pad at 50 degrees Celsius. Using a microscope in a lead-shielded set-up, the radio-active beads can be transferred with tweezers to their final positions. The phantom is constructed from a block of very light foam that is made adhesive on the top and bottom sides. The markers are placed close to the corners, and the adhesive sides are subsequently covered by a thin layer of foam. Markers can be semiautomatically extracted through maximum intensity projection (MIP) images in three directions. The location of each individual marker is determined by calculating its center of mass, denoted by *M*_*c*_. To determine the coregistration accuracy, we flipped the registration phantom vertically to make a different phantom as that changes all the markers to different positions.

### 2.5. Matching Algorithm

To find the transformation between the SPECT and the CT coordinate systems, Horn's quaternion-based algorithm was used [22, 23]. This algorithm provides a single-step solution to the rigid transformation between two coordinate systems given measurements of the coordinates of a set of points that are not collinear. The two sets of center of masses of the point markers in the registration phantom, as imaged in CT and in SPECT, were used to compute the transformation. A rigid body transformation was chosen as the voxel size of the micro-SPECT images, determined by the motion of an accurate *X* *Y* *Z* stage, is uniform and precisely known and the variation of the CT voxel size in the micro-CT images can be neglected (see Section 2.3).

### 2.6. Circular Cone-Beam CT Data Stitching

In circular cone-beam Micro-CT, it is likely that the length of the FOV of a single acquisition is shorter than the total length of the object, such as a rat in the case of preclinical application of Micro-CT. This leads to multiple acquisitions in different bed positions with translations in between, which can be provided by a motorized carriage. 

Without loss of generality we explain two different stitching methods by stitching three subvolumes with two stitching positions. Traditionally, all subvolumes scanned at different z-positions can be stitched by calculating translation positions and the CT voxel size. We scanned three subvolumes sequentially at three different z-positions, that is, Scan1, Scan2, and Scan3 with two overlapping stitching regions, Stitch1 and Stitch2. To minimize degrading effects due to the cone-angle, the voxel value within the overlapping region is chosen from that subvolume in which the position of this voxel is closest to center of the FOV. To obtain the stitched volume with minimal voxel mismatch, there are at least three prerequisites using this stitching method: (1) precisely known voxel size in the z direction, (2) precisely known bed translation distance between two adjacent scans, and (3) the bed must be translated in a fixed direction. If this direction is not perpendicular to the scanning gantry, its orientation needs to be precisely measured in order for the transaxial translation to be known. 

**Figure 5:**
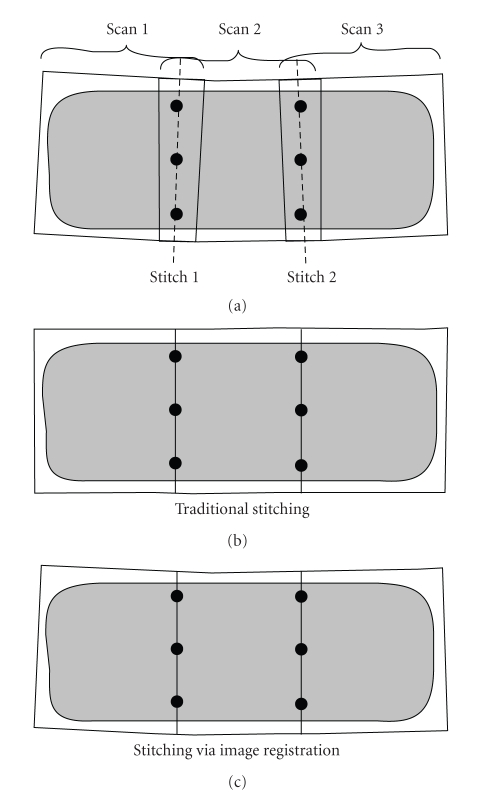
Diagram comparing traditional stitching and stitching via image registration. Given three scans (a) with overlap of an object with slightly different direction due to mechanical inaccuracies, traditional stitching will give a result as shown in (b), while stitching via image registration, in which the differences in direction are accounted for, will result in (c).

Practically, due to limited mechanical accuracy these three conditions are likely to be not completely satisfied. In order to reduce the resulting error from a small, but noticeable discontinuity in object shape to something virtually unnoticeable, we employed the same technique as was used in the SPECT-CT coregistration: a precalculated spatial transformation using a calibration phantom at the stitching position; see Figure 5. The calibration phantom closely resembled the registration phantom as shown in Figure 4. The size of the phantom was chosen so that it fully covers the maximum object size. Extra-point markers were added in the overlapping parts of the different acquisition bed positions. The spatial transformation between two adjacent subvolumes was calculated only once with Horn's algorithm. Once all transformation matrices are obtained, all subsequent reconstructed subvolumes imaged at these fixed positions can be quickly, accurately, and automatically combined using these precalculated matrices. After the application of the matrices, the volumes are sufficiently aligned so that the slices from the different subvolumes can be stitched together. All subvolumes scanned subsequently will be automatically stitched using these precalculated transformation matrices. After the application of the matrices, the volumes are sufficiently aligned so that the slices from the different subvolumes can be stitched together. For instance in Figure 5, scan1 (the moving volume) is stitched to scan2 (the fixed volume) by a transformation matrix calculated by the point markers at stitch1. Scan3 (the moving volume) is stitched to scan2 (the fixed volume) by a transformation matrix calculated by the point markers at stitch2.

**Figure 6:**
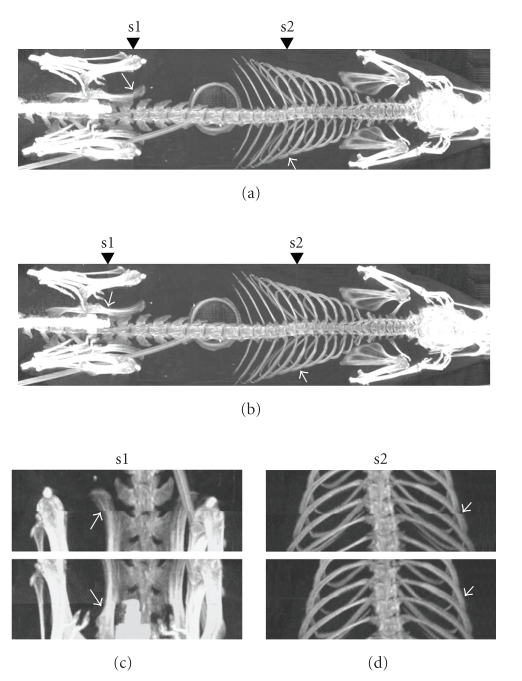
MIP images of the stitched rat X-ray CT volumes, comparing two stitching methods. (a) Conventional stitching. (b) Stitching by image registration. (c) Details at stitching positions, s1 and s2. Details where at the stitch location, the image either shows a mismatch or not, are shown by arrows.

Small animal scans were used to test the effects of two stitching methods. A 368 g rat was anesthetized using isoflurane during scanning at three bed positions (*Z* = 0, 65, and 130 mm), after which the volume of the entire rat was obtained separately using two different stitching methods.  Figure 6 shows an MIP image of a stitched rat volume (from top to bottom) by two stitching methods. There are relatively large voxel mismatches at the stitch positions using the traditional method. The stitching method via image registration can significantly lower the voxel mismatches and provides a good solution to voxel mismatching caused by limited FOV length in circular cone-beam CT.

### 2.7. SPECT-CT Registration Error Experiment

The reproducibility of the animal bed-mounting determines a large share of the total error of the coregistration between two modalities. We determined bed-mounting errors by calculating standard deviations of the center of mass *M*_*c*_ of each individual marker from a series of measurements in which the bed was remounted each time. The standard deviation of *M*_*c*_ in 3D is calculated by

(1) $$\sigma_{3\text{D}}=\sqrt{\frac{1}{N}\overset{N}{\underset{i=1}{\sum }}\left[{\left(X_{i}-\overset{̅}{X}\right)}^{2}+{\left(Y_{i}-\overset{̅}{Y}\right)}^{2}+{\left(Z_{i}-\overset{̅}{Z}\right)}^{2}\right]},$$

whereby *N* is total number of measurements of CT or SPECT images.

The maximum bed-mounting error in 3D is calculated by

(2) $$\begin{array}{cc} E_{\max\;}^{3\text{D}} & =\max\;\;\left(\sqrt{{\left(X_{i}-\overset{̅}{X}\right)}^{2}+{\left(Y_{i}-\overset{̅}{Y}\right)}^{2}+{\left(Z_{i}-\overset{̅}{Z}\right)}^{2}}\right)\\ & \;\;\;\;\;\;\;\;\;\;\;\;\;\;\;i\in \left[1,N\right]. \end{array}$$

### 2.8. Coregistration Error Experiment

As the resolution of the CT images is at least four times higher than the SPECT images, we usually perform the transformation in the direction of reslicing and transforming the SPECT images to match the corresponding CT images. We calculated this transformation only once from a single CT and a single SPECT measurement and applied this transformation to other images acquired by mounting the bed multiple times. The coregistration errors in *X*, *Y*, and *Z* were calculated by

(3) $$E_{P}=\sqrt{\frac{1}{N}\overset{N}{\underset{i=1}{\sum }}{\left(P_{\text{SPECT}}^{i}-{\overset{̅}{P}}_{\text{CT}}\right)}^{2}}\;\left(P=X,Y,Z\right),$$

where *N* is the total number of measurements of SPECT images. The coregistration error in 3D was calculated by

(4) $$\begin{array}{cc} E_{3\text{D}} & =\sqrt{\frac{1}{N}\overset{N}{\underset{i=1}{\sum }}\left[{𝒜}^{2}+{ℬ}^{2}+{𝒞}^{2}\right]}, \end{array}$$

where *𝒜* denotes $X_{\text{SPECT}}^{i}{-\overset{̅}{X}}_{\text{CT}}$, *ℬ* denotes $Y_{\text{SPECT}}^{i}-{\overset{̅}{Y}}_{\text{CT}}$, and *𝒞* denotes $Z_{\text{SPECT}}^{i}-{\overset{̅}{Z}}_{\text{CT}}$. The maximum coregistration error in 3D is calculated by

(5) $$\begin{array}{cc} E_{\max\;}^{3\text{D}} & =\max\;\;\left(\sqrt{{𝒜}^{2}+{ℬ}^{2}+{𝒞}^{2}}\right)\\ & \;\;\;\;\;\;\;\;i\in \left[1,N\right], \end{array}$$

where *𝒜* denotes $X_{\text{SPECT}}^{i}{-\overset{̅}{X}}_{\text{CT}}$, *ℬ* denotes $Y_{\text{SPECT}}^{i}-{\overset{̅}{Y}}_{\text{CT}}$, and *𝒞* denotes $Z_{\text{SPECT}}^{i}{-\overset{̅}{Z}}_{\text{CT}}$.

### 2.9. Phantom Experiments for Registration Validation

A length of flexible plastic tube (approximately 10 cm) with an inner diameter of 0.4 mm was filled with a mixture of CT contrast agent and ^99 m^Tc. The two ends were cut and sealed using the sharp edge of a pair of heated tweezers. A helix phantom was formed by winding the thin tube around a cylindrical plastic object (hollow and rigid, diameter 10 mm) using adhesive tape.

In addition, a Jaszczak hot-rod microresolution phantom (VanderWilt Techniques, The Netherlands) was used to illustrate how the registration accuracy relates to the system resolution. The capillary diameters in the six different segments are 0.35, 0.40, 0.45, 0.50, 0.60, and 0.75 mm for testing mouse-sized object imaging and 0.7, 0.8, 0.9, 1.0, 1.2, and 1.5 mm for testing rat-sized object imaging. The distances between the capillaries in a segment are equal to the capillary diameter within that segment. With 550 MBq/mL of ^99 m^Tc, the phantoms were scanned for an hour. The phantoms were subsequently scanned in both CT and SPECT. The capillary phantom can illustrate both the resolution and the coregistration accuracy when SPECT and fused images are shown side by side.

### 2.10. Animal Experiments

Animal studies were conducted following protocols approved by the Animal Research Committee of the University Medical Center Utrecht. High-resolution mouse and rat scans were used to test the registration accuracy. A 30 g male C57Bl/6 mouse was injected intravenously with 725 MBq of [^99 m^Tc] hydroxymethylene diphosphonate (HDP) ([^99 m^Tc] HDP) and was anesthetized using isoflurane during scanning. One hour after HDP injection, the mouse was imaged for an hour in the micro-SPECT system using the mouse collimator tube with 0.35 mm diameter pinholes. This was followed by a Micro-CT scan. Similarly, a 368 g male Wistar rat was injected with 950 MBq of [^99 m^Tc] HDP. Three hours later SPECT data were obtained for two hours using 1.0 mm diameter pinholes. Micro-CT data was acquired at three bed positions (*Z* = 0, 65, and 130 mm), after which the volume of the entire rat was obtained automatically by means of 3D stitching, as mentioned in Section 2.6. It should be noted that we used a relatively high amount of activity for the above scans. This was because the highest possible SPECT resolution was required for the testing.

## 3. Results

### 3.1. Bed-Mounting Error

Table 1 shows the statistics of standard deviation of *M*_*c*_ (*N* measurements) analyzed for different markers located at different corners on the testing object. In this table, the value of “Average” is the average of standard deviation of *M*_*c*_ over all markers, while the value of “Maximum” is the maximum of the standard deviation of *M*_*c*_ for all markers.

Table 2 shows statistics of maximum bed-mounting error (*N* measurements) analyzed for all markers. 

### 3.2. SPECT-CT Coregistration Error

Table 3 shows the statistics of coregistration error analyzed for markers at different locations. In this table, the value of “Average” is the average of coregistration error (*N* measurements) over all markers, while the value of “Maximum” is the maximum coregistration error of all markers.

Table 4 shows statistics of coregistration error (different measurements) analyzed for all markers. For reasons of comparison, the coregistration error was also calculated for the rat bed in which the volumes were stitched using the traditional method (numbers between brackets).

### 3.3. Phantom Experiments

**Figure 7:**
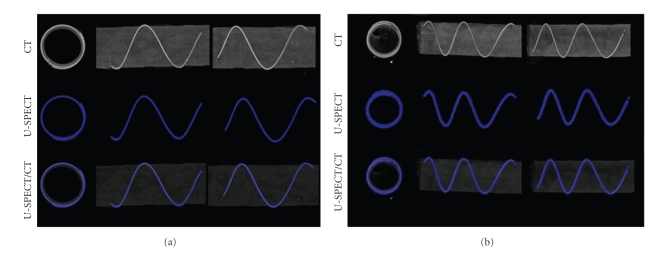
MIP images of helix phantom illustrate registration accuracy in three directions. (a) SPECT imaging with mouse collimator. (b) SPECT imaging with rat collimator. Top row: CT image, middle row: SPECT image, and bottom row: fused SPECT-CT image.

Figure 7 shows MIPs of the helix phantom from three different directions representing the mouse (frame (a)) and rat (frame (b)) imaging. The top row shows CT images and the central row SPECT images. The fused images on the bottom row show that the profile of the helix tube in the CT image (bright) is situated entirely along the center line of its corresponding SPECT region (blue). In the case of the rat tube, the air bubble and the liquid behind it at the end of the tube are also well matched as shown by the arrow on Figure 7(b).

**Figure 8:**
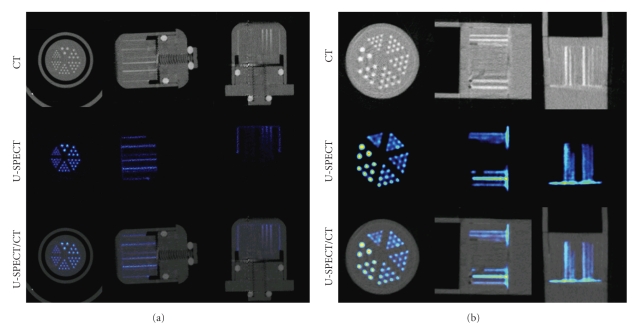
The capillary resolution-phantom shows that the registration does not mismatch more than the smallest capillaries visible in SPECT. The capillary diameters in the six different segments are 0.35, 0.40, 0.45, 0.50, 0.60, and 0.75 mm for mouse imaging and 0.7, 0.8, 0.9, 1.0, 1.2, and 1.5 mm for rat imaging. (a) SPECT imaging with the mouse collimator, (b) SPECT imaging with the rat collimator. Top row: CT image, middle row: SPECT image, and bottom row: fused SPECT-CT image.

Figure 8 shows MIP images, taken from three directions, of a capillary resolution phantom for both the mouse and rat collimator. The area around the hot rods and the cylindrical space between the insert and the inner-wall of the capsule show a good match between CT and SPECT. The capillary resolution-phantom illustrates that the registration does not mismatch more than the smallest capillaries visible in the high resolution SPECT images.

### 3.4. Animal Images

**Figure 9:**
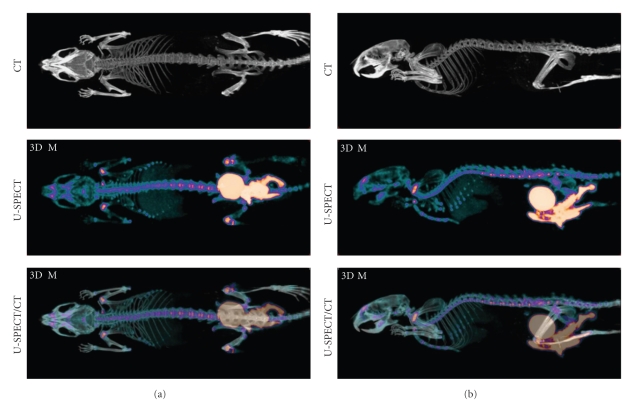
MIP images of registered total-body mouse scans. Top row: CT image, middle row: SPECT bone image, and bottom row: fused SPECT-CT image.

**Figure 10:**
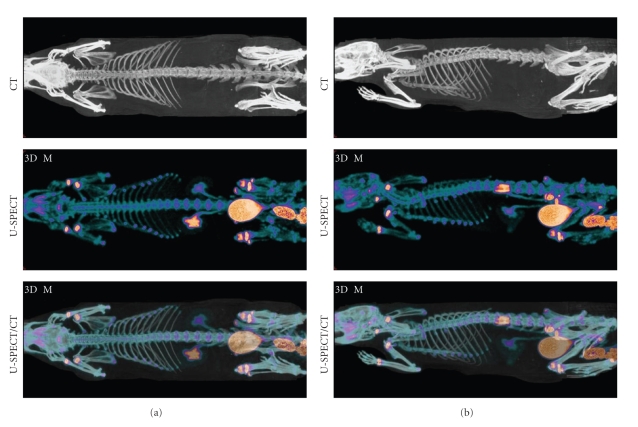
MIP images of co-registered total-body rat scans. Top row: CT image, middle row: SPECT bone image, and bottom row: fused SPECT-CT image.

Maximum Intensity Projections (MIPs) of a total-body mouse and rat bone scan at two different orientations are shown in Figures 9 and 10. As we can see, these images indicate good matching between CT and SPECT images. As in the phantom images, the small details of the SPECT images such as the ribs overlap nicely with the CT images in spite of the expected animal motion because of breathing.

## 4. Discussion

In this paper, rat- or mouse-sized registration phantoms with point sources as markers are used to find the spatial relation, represented by a precalculated transformation matrix, between separate modalities. The specially designed multimodal animal bed, which is transferable, can be rigidly, rapidly and reproducibly mounted to CT and SPECT scanners. Both phantom and animal images illustrate the effectiveness of the method. Average overall coregistration accuracies in 3D are below 0.24 mm for mouse imaging and below 0.42 mm for rat imaging. This accuracy is better than the image resolution of the ultra-high-resolution SPECT used in this study and was confirmed by a good overlap of the smallest visible details in phantom and animal images acquired in this study. Stable animal position during bed-transfer and scanning is a pre-requisite for image coregistration using a precalculated transformation.

Our results show also that extending the field-of-view for micro-CT images with prior registration of the volumes gives improved results over conventional stitching. In this way rat-sized objects can be scanned in the relatively small FOV of the micro-CT without stitching errors. Additionally the coregistration errors for registration between CT and SPECT volumes for rat-sized objects are also reduced when prior registration of the CT volumes is used (see Table 4).

Another possible method to obtain the coregistration information is to use multimodal fiducial markers during each image acquisition [24, 25]. The advantage of this is that it is relatively straightforward to do and that there are several 3D image processing software packages that support it well. However, it also has disadvantages. First of all, there must always be sufficient space to accommodate the additional fiducial markers in the animal bed. Secondly, it often requires manual steps to identify the marker locations for each individual scan to be co-registered. Thirdly, markers can cause image artifacts, particularly in SPECT. Furthermore, because markers are on the outside of the animal, the total imaged volume needs to be increased from only the organs of interest to a volume that includes the markers. If highly focusing SPECT devices need to cover a larger volume, this reduces the count yield from the actual organs of interest. 

In our approach we used Horn's quaternion-based algorithm to compute the transformation matrix based upon scans of the registration phantom [22, 23]. Horn's algorithm is a noniterative closed-form solution of the absolute orientation problem. Iterative solutions for the absolute orientation have some drawbacks. For instance, they only provide an approximation of the least-squares solution, selectively neglecting constraints, are generally computationally intensive, and rely on initial transformation parameters [26]. Horn's Algorithm can instantly calculate the transformation matrix. It should be noted that Horn's algorithm relies on exact point-to-point correspondence to determine the transformation. However, this will not pose a problem for us as there is always good apriori orientation between the two different imaging modalities. 

For animal imaging, the sources of inaccuracy will include the movement of the animal. If the final accuracy is not limited by the inaccuracy introduced by transferring the bed, but by motion that is always present, such as breathing, then it can be concluded that the approach with transferable beds has tolerable errors and is suitable for independent small-animal scanners, or comparable to combined SPECT-CT scanners, as their images are usually also acquired sequentially with a robot translation in between. 

## 5. Conclusion

We presented an accurate method for coregistration of sub-half-mm resolution small-animal SPECT images with X-ray CT images. In addition we showed that extending the FOV of cone-beam CT by stitching is improved by prior registration of the CT volumes. We showed that the registration error is typically much smaller than the image resolution of high-end SPECT devices. Therefore, this approach of coregistration using a transferable bed can be a good alternative to integrated multimodality systems. The method eliminates the necessity for the laborious manual registration of multimodality images and opens possibilities to perform multimodality imaging of anesthetized animals without integrated systems. Validation for other tomographic modalities such as MRI will be a subject of further research.

## Figures and Tables

**Table 1:** Average and maximum of standard deviation of *M_c_* analyzed for different markers.

	Standard deviation (mm)	Mouse bed (Φ0.35 mm pinholes)		Rat bed (Φ1.0 mm pinholes)	
		Average	Maximum	Average	Maximum
CT	*X*	0.0400	0.0617	0.0869	0.1335
	*Y*	0.0869	0.1120	0.0777	0.1358
	*Z*	0.0883	0.1016	0.1314	0.1666
	3D	**0.1329**	**0.1553**	**0.1806**	**0.2273**
		3 mountings, 5 markers		4 mountings, 8 markers	

SPECT	*X*	0.0280	0.0364	0.0188	0.0392
	*Y*	0.0428	0.0548	0.0269	0.0474
	*Z*	0.0398	0.0526	0.0210	0.0342
	3D	**0.0667**	**0.0716**	**0.0415**	**0.0590**
		4 mountings, 5 markers		4 mountings, 8 markers	

**Table 2:** Statistics of maximum bed-mounting error analyzed for all markers.

	Maximum error(mm)	Mouse bed (Φ0.35 mm pinholes)		Rat bed (Φ1.0 mm pinholes)	
		Average	Maximum	Average	Maximum
CT	*X*	0.0483	0.0777	0.1308	0.2066
	*Y*	0.1095	0.1582	0.1074	0.1783
	*Z*	0.1169	0.1392	0.1627	0.2322
	3D	**0.1497**	**0.1901**	**0.2229**	**0.2697**
		3 mountings, 5 markers		4 mountings, 8 markers	

SPECT	*X*	0.0320	0.0500	0.0252	0.0541
	*Y*	0.0489	0.0590	0.0410	0.0802
	*Z*	0.0459	0.0690	0.0260	0.0569
	3D	**0.0842**	**0.0947**	**0.0575**	**0.0979**
		4 mountings, 5 markers		4 mountings, 8 markers	

**Table 3:** Average and maximum of deviation analyzed for different markers.

Deviation (mm)	Mouse bed (0.35 mm pinholes)		Rat bed (1.0 mm pinholes)	
	Average	Maximum	Average
*X*	0.0435	0.0736	0.2167	0.5594
*Y*	0.1044	0.1215	0.1400	0.2752
*Z*	0.1944	0.3317	0.2756	0.5273
3D	**0.2394**	**0.3512**	**0.4190**	**0.6312**
	5 markers		8 markers	

**Table 4:** Statistics of maximum coregistration error analyzed for all markers.

Coregistration error (mm)	Mouse bed (0.35 mm pinholes)		Rat bed (1.0 mm pinholes)	
	Average	Maximum	Average	Maximum
*X*	0.0561	0.0923	0.2127(0.3045)	0.5632(0.6702)
*Y*	0.1017	0.1777	0.1286(0.4048)	0.2979(0.5816)
*Z*	0.0749	0.1991	0.1440(0.3668)	0.3964(0.6299)
3D	**0.2843**	**0.3779**	**0.4448**(0.8672)	**0.6481**(1.0880)
	(5 markers)		New stitching (traditional stitching) (8 markers)	
